# Should we use philosophy to teach clinical communication skills?

**DOI:** 10.4102/phcfm.v8i1.1292

**Published:** 2016-11-16

**Authors:** Berna Gerber

**Affiliations:** 1 Division of Speech-, Language- and Hearing Therapy, Faculty of Medicine and Health Sciences, University of Stellenbosch, South Africa; 2 Department of Philosophy, Faculty of Arts and Social Sciences, University of Stellenbosch, South Africa

## Abstract

Effective communication between the doctor and patient is crucial for good quality health care. Yet, this form of communication is often problematic, which may lead to several negative consequences for both patients and doctors. Clinical communication skills have become important components of medical training programmes. The traditional approach is to teach students particular communication skills, such as listening to patients and asking open-ended questions. Despite their importance, such training approaches do not seem to be enough to deliver medical practitioners who are able and committed to communicate effectively with patients. This might be due to the pervasive negative influence of the medical profession’s (mistaken) understanding of itself as a natural science on doctor–patient communication. Doctors who have been trained according to a positivist framework may consider their only responsibility to be the physical treatment of physical disorders. They may thus have little regard for the patient’s psychological and social world and by extension for communication with the patient and/or their caregivers. To address this problem, I propose a curriculum, based on the academic field of philosophy, for teaching clinical communication.

## Introduction

Doctor–patient communication is central to the practice of medicine.^1,2,3^ Communication skills form a core component of a doctor’s clinical competence, alongside knowledge and skills in problem-solving and physical examination.^4^ Many of the tasks of the consultation are accomplished through and assisted by communication with the patient, such as arriving at a diagnosis and reaching the ethical ideal of shared decision-making.^5,6^ Unfortunately, a vast body of research literature indicates that doctor–patient communication is often problematic.^4^ The greatest problems seem to be that sufficient communication seldom takes place between the doctor and patient and that doctors commonly do not regard communication as important to their clinical work. Poor doctor–patient communication may have many negative consequences for both patients and doctors, such as poor patient outcomes, low patient satisfaction, lower adherence to prescribed treatment and malpractice lawsuits against doctors.

## Clinical communication skills training

It is perhaps for these reasons that clinical communication skills are becoming more important within medical curricula. The traditional approach is to teach students particular communication skills, such as listening to patients and asking open-ended questions. Although these skills are important to include in clinical communication courses, they are – by themselves – seldom successful in producing medical practitioners who are able and committed to communicate effectively with patients.^7,8^

It seems that something might be missing from the research about doctor–patient communication. Many other factors have been identified as influences on doctor–patient communication and their effects have been studied and documented, such as the context of the interaction and the personal characteristics of the doctor and the patient. Yet, one serious question that is seldom addressed is the question about the influence of medicine’s positivist view of the world and of itself on communication between two persons, a doctor and a patient, who are engaged in a very *human activity*, namely attempting to identify and treat disease and to lessen the suffering that comes with it.

## Modern medicine’s intellectual self-image

For at least the past 100 years, medical professionals have regarded medicine to be a natural science.^3,9,10^ The understanding of science among the medical fraternity closely corresponds with the logical positivist view of science.^9^ Positivism refers to the view ‘… that the highest or only form of knowledge is the description of sensory phenomena’.^11^ According to this tradition, our knowledge of the world results from observation of the senses.^12^ For a statement to have a claim to truth, it needs to be possible to verify the statement by means of sensory experience.^13,14^ Statements that cannot be empirically verified are not regarded as true or meaningful.^13,14^

I argue that medicine’s positivist world view may be an important reason for the stubbornness of clinical communication to conform to patient-centred ideals. Positivism leads to an almost exclusive focus on the physical aspects of disease in clinical medicine. The patient’s mind and social world are not of great significance within this perspective. Medical professionals who have been educated according to a positivist framework may easily regard their clinical task solely as the physical treatment of physical disorders. They are, therefore, very likely to consider many communicative activities as unrelated to their clinical task.^15^

## Is the intellectual self-image of medicine appropriate?

Many scholars^1,2,3,9,16,17,18,19^ have pointed out that clinical medicine in itself is not a natural science (at least not in the positivist sense). It is best described as the *practice* of caring for the sick, treating their disease and of preventing (further) disease and disability. Kathryn Montgomery^3^ describes medicine as ‘a learned, rational, science-using practice’ and science as a tool instead of the soul of clinical medicine. To perform their clinical tasks, the doctor needs knowledge of the biomedical sciences, adequate clinical experience and skills, as well as knowledge of the vagaries of the human condition.^3^

## The implications for clinical medicine and medical education

Educational interventions that aim to improve doctor–patient interactions are unlikely to have much or lasting success while doctors are taught to approach their clinical work in a positivist manner. What is ultimately needed is a transformation of the medical profession’s intellectual self-image and world view to better align with the nature of clinical practice and to make room for the psychological and social dimensions of the patient’s life within health care. To accomplish such a paradigm shift, room should be made for the human and social sciences in medical education and research.^20^ Furthermore, the medical profession needs to revise its conception of science. Modern medicine’s positivist conception of science should be replaced by an intellectually more robust and updated theory of science. Such a theory will acknowledge that interpretive reasoning, that is, characteristic of the way that doctors think when they practice medicine, and knowledge without guaranteed certainty are legitimate elements of science.^19^

To address many of the persistent problems in doctor–patient communication, I propose a curriculum informed by the field of philosophy. Such a curriculum can be presented to qualified medical practitioners and registrars, for instance, in the form of continued professional development activities, and also to undergraduate medical students.

## A proposed curriculum for clinical communication

The broad aims of a clinical communication course based on philosophy would be (1) to foster among the participants an appreciation of the nature and identity of clinical medicine as a particular professional discipline and praxis and (2) to develop an understanding of what such self-knowledge means for (effective) communication and good relationships with patients and their caregivers. More specifically, attention can be paid to the concepts and ideas described below.

## Reflection on the concept of science

The prevalent and problematic logical positivist view of science among medical practitioners should be interrogated. Discussions should be had about the problems associated with the view that scientific knowledge is absolutely objective, that is, a reflection of the nature of reality as it truly is, unclouded by the personal reactions of the person involved in the scientific activity to that which he experiences,^21,22,23^ or by his social circumstances or historical context, or by theories or ideologies in his mind.^14^

## The goal of clinical medicine and how it differs from the goal of general (biomedical) science

The goal of clinical medicine is to diagnose and treat the diseases suffered by particular individuals. In contrast, the goal of the biomedical sciences is to create knowledge that enlarges and solidifies our scientific understanding of health and disease in general.^19^ The goals of scientific reasoning are precision and predictability, whereas the reasoning of the doctor tending to their patient is aimed at finding the best possible answer under the particular circumstances.^3^

## An analysis of three central concepts of clinical medicine

These concepts are ‘patient’, ‘disease’ and ‘therapy’.^9^ Medical curricula should include a critique of the meaning of these concepts in purely positivist terms, especially in view of the possible negative implications of such understandings for meaningful relationships and effective communication between doctors and their patients. In particular, attention should be paid to the nature of patients as unique individuals who possess rationality and how this qualitatively distinguishes them from the relatively stable and simple physical phenomena, such as molecules, that are studied by certain natural sciences. Almost everything that we want to know about such simpler forms of life can be answered by referring to the chemical and physical mechanisms that afford them their continuing existence and characteristics. When scientists make generalisations about the behaviour of such simple physical matter, this knowledge is usually perfectly reliable^24^ and may be regarded as ‘certain knowledge’.^3^ This certainty stems from the fact that very little diversity exists between simpler phenomena. Every instance of a molecule of a certain kind, such as a molecule of water (H_2_O) or sodium chloride (NaCl), is very much like any other instance of the same kind of molecule.^24^ By comparison to the relative uniformity of simple life forms, and as a constant frustration to doctors, individual human patients are unique in terms of their biology as well as their biography and psychology. Doctors know that not all patients who suffer from the same disease have the same set of symptoms. Moreover, treatments that are effective in some or even most patients with a particular condition may be ineffective or harmful in others. Because patients are unique and have the capacity to reason and the right to make the decisions about the health care they receive, doctors *need to* communicate with them in order to practice effective and ethical health care. This idea closely relates to Greenhalgh et al.’s^25^ plea for clinical practice driven by patient-centred evidence.

## The nature of clinical rationality (or how do doctors think?)

Doctors reason in an interpretive, dialogical and narrative manner when they treat patients.^1,3,19^ This is different from the deductive way in which natural scientists reason, from the general to the specific.^3,18^ Doctors reason ‘from the particular to the general and then (for confirmation) back again’.^3^ Doctors cannot directly apply their general scientific knowledge of biological laws and facts, such as pathophysiology, to identify and treat disease in individual patients. This is because biological laws are imprecise and abstract and individual patients are unrelentingly unique.^3,19^

After instruction and reflection on these curricular themes, medical students and practitioners should be guided to consider what such a heightened and more accurate sense of ‘professional intellectual identity’ means for (effective) communication and relationships with patients and caregivers. A ‘stand-alone’ course based on philosophy might not be enough to bring about meaningful and long-standing change in the way that doctors think about their work and about the clinical importance of effective communication with patients. Such change can only be imagined when a transformed and appropriate self-image has been formulated and becomes integrated into all of the courses that medical students and registrars need to complete.

## Conclusion

In response to the persistent and widespread problem of poor quality doctor–patient communication, I propose a non-traditional approach to clinical communication education. This approach is aimed towards revising the dominant and mistaken self-understanding of medicine in natural scientific terms. It is envisioned that doctors who have an appropriate non-positivist conceptualisation of the nature of clinical medicine and its central components – patient, disease and therapy – will appreciate and give expression to the necessity of effective communication with patients for quality health care.
